# Socio-Economic Factors, the Food Environment and Lunchtime Food Purchasing by Young People at Secondary School

**DOI:** 10.3390/ijerph16091605

**Published:** 2019-05-08

**Authors:** Wendy Wills, Giada Danesi, Ariadne Beatrice Kapetanaki, Laura Hamilton

**Affiliations:** 1 Centre for Research in Public Health and Community Care (CRIPACC), University of Hertfordshire, College Lane, Hatfield, Herts AL10 9AB, UK; 2 Faculty of Social and Political Sciences, University of Lausanne, UNIL-Mouline, Bâtiment Géopolis, 1015 Lausanne, Switzerland; giada.danesi@unil.ch; 3 Department of Marketing and Enterprise, Hertfordshire Business School, University of Hertfordshire, Hatfield, Herts AL10 9AB, UK; a.b.kapetanaki@herts.ac.uk; 4 Thomas Coram Research Unit, UCL Institute of Education, 27–28 Woburn Square, London WC1H 0AA, UK; l.hamilton@ucl.ac.uk

**Keywords:** food purchasing practices, food environment, secondary school, young people, SES, mixed methods, qualitative methods, purchasing recall questionnaire

## Abstract

The aim of this paper is to report on the lunchtime food purchasing practices of secondary school students and some of the factors related to this purchasing, including the influence of socio-economic status (SES) and the food environment within and around schools. A mixed-methods study incorporating an online purchasing recall questionnaire and multiple qualitative methods was undertaken at seven UK secondary schools. The analysis shows that SES was intricately woven with lunchtime food practices. Three-quarters of participants regularly purchased food outside of school; those at low SES schools were more likely to report regularly leaving school to buy food. Young people’s perception of food sold in schools in areas of low SES was often negative and they left school to find “better” food and value for money. Taste, ingredients and advertisements were factors that mattered to young people at schools with low or mixed SES; health as a driver was only mentioned by pupils at a high SES school. For public health initiatives to be effective, it is critical to consider food purchasing practices as complex socio-economically driven phenomena and this study offers important insights along with suggestions for designing interventions that consider SES. Availability of food outlets may be less important than meeting young people’s desires for tasty food and positive relationships with peers, caterers and retailers, all shaped by SES. Innovative ways to engage young people, taking account of SES, are required.

## 1. Introduction

At a time when there is a global appetite for investing in the health and nutritional wellbeing of young people [1], there is a need to better understand young people’s food purchasing practices during the secondary school day [2]. Interventions that ignore these practices are perhaps unlikely to achieve nutritional improvements at the population level [3,4]. Socio-economic deprivation is associated with consuming a poorer diet, with young people living in low income areas often consuming fewer fruits and vegetables and more food and drinks containing sugar, when compared to children from more affluent areas [5]. The number and type of food businesses in the vicinity of schools is also considered to be an important factor and this is likewise linked to the socio-economic status (SES) of an area [6]. Whilst in some countries it is common for young people to eat lunch that is prepared and sold within schools [7] and which meets regulated nutritional standards, in other countries, including the USA [8,9] and UK, eating practices vary. Young people can bring food and drink from home to consume at school, some purchase and consume food or drink bought from local retailers, some go home to eat lunch, others do not eat or drink during the school day some pupils stay on site to purchase food or drink. This degree of “choice” means that those working to improve public health need to know more about the food purchased and consumed inside and outside of school and the factors relating to these practices in order to develop effective intervention strategies.

A third of young people’s daily energy intake is consumed during the school day, so this setting substantially contributes to overall energy intake [10]. Eating habits established during childhood or adolescence are likely to be sustained into adulthood thereby adding to the need to encourage the consumption of a nutritious diet during secondary school [11]. In Scotland, the area of the UK where the study drawn on in this paper was conducted, there is a commitment to assess whether measures to restrict access to food and drink in the area around schools is more likely to result in improved nutritional choices for young people or whether schools remain the site where intervention should be focused [12,13].

The aim of this paper is to describe the lunchtime food purchasing practices of young people aged 13–15 years and to shed light on the factors related to this purchasing, including the influence of SES and the food environment within and around schools. This paper also provides an overview of what food is purchased by young people and from what type of food businesses. These results will help to guide future interventions that seek to improve the overall nutritional quality of food practices and address obesity levels among young people [11] in countries where diet and obesity are a public health concern. 

## 2. Materials and Methods

The study uses a mixed methods approach to enable the complex social phenomena of food purchasing within and beyond the school gate to be described, explored and explained without losing sight of the overall context of the food environment or the influence of SES. Mixed-methods studies allow multiple, dynamic factors to be described and explored (different people, in different settings, doing different things, at different times) [14,15]. We used a convergent, parallel mixed-methods design whereby the quantitative and qualitative components of the study were valued equally and carried out concurrently [14,16]. Seven secondary schools were purposefully recruited across Scotland so that we could include schools in areas of varying SES and food outlet type/density. Local authorities were approached to help identify potential schools before Head Teachers in areas of varying SES and food outlet type/density were contacted to ask for consent for their school to participate. Eight-hundred metres was chosen as the radius to look at food outlet type/density around schools as this represents a 10-min walk (feasible during a 40–60-min lunch period); this distance was also used in another study of food businesses around Scottish schools [17]. We determined the density/type of food outlets around schools by accessing local authority details of registered food businesses, supplemented with fieldwork observation of food outlets and young people’s annotations of maps provided during the semi-structured written exercise in class (for further details of how information was gathered about food outlets please see [18]. SES was determined based on the Scottish Index of Multiple Deprivation (SIMD) rankings of school postcodes and the proportion of students registered for means-tested free school meals (FSM) (Based on parents’ income and/or government benefits received. Families with the lowest incomes are eligible for free school meals).

This study was conducted according to the guidelines laid down in the Declaration of Helsinki and all procedures involving human subjects were approved by the Health and Human Sciences Ethics Committee with Delegated Authority at the University of Hertfordshire (Ref: HSK SF/UH/00045). Written consent was obtained from all young people who contributed to qualitative parts of the study. Completing the online questionnaire in the presence of the research team was taken as young people’s assent to participate in this part of the research. Verbal consent from head teachers, kitchen supervisors and retailers was obtained and witnessed. Parents of young people at the participating schools were sent information about the study at the outset and could “opt out” their child if they wished to (none did so). 

### 2.1. Quantitative Methods and Data Analysis

A purchasing recall questionnaire (PRQ) was developed, to ask young people in detail about their lunchtime food and drink purchasing outside school (what was bought and from where) and the importance of marketing initiatives and other factors that might influence their purchasing habits. Information was also sought about the frequency of food or drink purchasing outside school and whether anything was consumed before school or during mid-morning break. 

To ensure that the PRQ was valid and reliable, items regarding the “reasons for visiting outlets” were borrowed and adapted to the purposes of this study from Chowdhury et al. [19] and Swoboda et al. [20] and the items regarding “factors important when food/drink purchased at lunchtime” has been designed to include all the marketing elements that can have an impact on young people’s purchasing habits, based on relevant marketing and food/ healthy eating literature. The food and drink items included on the PRQ were adapted from the “Food and Drink on School Days” questionnaire (version 10) [21]. The adapted categories were compared with food frequency questionnaires for this age group from validated dietary assessment tools (http://www.noo.org.uk/core/frameworks/SEF_Diet; http://dapa-toolkit.mrc.ac.uk/). Additional food or drinks identified from these sources (namely yogurts and milk-based desserts) were added to the PRQ. The PRQ was piloted with the pupils of one secondary school. Six classes with 145 students (68 students aged 13–14 years and 77 students aged 14–15 years) participated in a pre-pilot then pilot study. The overall aim of the pre-pilot and pilot studies was to test and refine the methods and fieldwork information/documents ahead of the main study and to involve young people in refining the study design and its implementation.

The PRQ was, where possible, administered online immediately following the lunch period [22] via PCs in classrooms; members of the research team were present in order to assist with any queries or difficulties when young people were completing the questionnaire. The PRQ data were exported from the online questionnaire software into SPSS (version 20, SPSS Inc., Chicago, IL, USA). A descriptive analysis of the PRQ data is presented in this paper in combination with the qualitative analyses.

### 2.2. Qualitative Methods and Data Analysis

The qualitative part of the study involved observations of the food environment and food purchasing practices in schools, of the local food environment and of pupils’ food purchasing practices outside school. Members of the research team wrote field notes and took photographs of the observations they made. Semi-structured interviews [23] were conducted with head teachers, kitchen supervisors, retailers and young people. Interviews were audio recorded and transcribed. Interviews with young people were often conducted after some had taken the research team on “go-along” tours [24] of the school or the local area, to ascertain why they made certain purchases. Interviews with school staff were used to explore the school’s food policy and perceptions relating to why young people do or do not stay in school to purchase food. The retailers were asked about marketing initiatives, in terms of price, promotions, products and the outlet environment [25] as well as their perceptions about their customers [26] and what they buy, drawing from relationship marketing theory in the services context [27]. Two focus groups were conducted within each school, one with young people aged 13–14 and one with 14–15 years olds. Focus groups were video and audio recorded to ensure playback was of a sufficient quality to discern what participants said. Photographs taken by the research team were used to elicit discussion during focus groups with young people about local food retailers and the food service available in school. Young people who completed the online PRQ were also invited to complete a semi-structured written exercise, whereby they could write, prompted by statements written by the research team, about where they purchased food from and why and what their family felt or knew about their food purchasing. 

The constant comparative analysis involved several stages, including importing transcripts from interviews/focus groups, field notes and data from the semi-structured written activity into QSR NVIVO for Windows (Version 10), then repeatedly reading through these data to look for and code emergent themes or categories [28]. Video and audio recordings of focus groups and photographs taken during fieldwork were also viewed/listened to and notes on themes from these data were also written up in Word documents and coded in NVIVO. In line with a convergent, parallel mixed method study, analysis of the qualitative data was conducted independently from the quantitative analysis and then the findings from both components were used to address the overall study aim [16]. 

## 3. Results

Overall 651 young people took part in the qualitative sections of the study and 535 young people completed the PRQ (see Table 1). Six head teachers and seven kitchen supervisors were interviewed plus 25 retailers who managed, owned or worked in supermarkets and grocery stores; chain and independent bakeries; chain and independent takeaway outlets; independent cafes; food vans and leisure or community centres.

### 3.1. Description of Participating Schools 

The participating schools varied in terms of the number of students attending, SES and the proportion of students registered for means-tested free school meals (FSM) (see Table 2). Four of the schools had low SES and one school had high SES, according to the SIMD decile rank. The other two schools (Sch04 and Sch07) had higher than average numbers registered for FSM; fieldwork observations showed that the catchment area around these two schools (where young people lived) could be classified as being of lower as well as higher SES. Schools 04 and 07 are hereafter described in this paper as being in areas of ‘mixed SES’.

### 3.2. The Food Environment around Participating Schools

Schools differed in terms of the density of food businesses located within 800 m, from five outlets near to Sch06 to 249 outlets near to Sch05 (Table 3). The two schools with the lowest density (*n* = 0–20) of food outlets (Sch02; Sch06) were classified as having low and high SES, respectively, therefore no straightforward conclusions can be drawn about the relationship between SES and food outlet density from this study. Only a small number of registered food businesses (*n* = 77) were visited by young people observed or participating in the study, out of a total of 489 registered/observed food outlets. Table 3 highlights the different type of food outlets that were located within 800 m of each school. 

### 3.3. Eating or Drinking before School, at Mid-Morning Break and at Lunchtime

To contextualise the results, we first describe the proportion of young people who ate or drank something before school, at mid-morning break and at lunchtime. More than six in ten young people reported on the PRQ that they had something to eat at home before school (62.8%). Seventeen percent said they did not eat or drink anything before school on the day they were surveyed and 12.5% of students reported consuming a drink only; for some of these young people this was reported to be a glass of water. Almost a quarter of students at Sch01 (low SES), Sch04 (mixed SES) and Sch07 (mixed SES) said they ate/drank nothing before school or consumed only a drink whilst only 6.6% at Sch06 (high SES) said they ate/drank nothing before school. At mid-morning break, across all schools, 58.9% of young people reported eating something and 65.0% said they had something to drink. At Sch07 (mixed SES) 59.5% said they did not eat anything at mid-morning break, a considerably higher proportion of pupils than at other schools. On the day that the PRQ was administered, 24.3% and 19.1% of young people said they did not eat or drink anything at lunchtime respectively. 

### 3.4. Leaving School at Lunchtime 

In the PRQ, 77% (*n* = 412) of young people said they left school to purchase food or drink at least twice a week (classified by the research team as “regularly” purchasing outside school at lunchtime). The proportion of young people from each school regularly purchasing food or drink beyond the school gate differed. The smallest percentage of students reporting going out regularly was from Sch06 (42.6%), the least deprived school studied, with a low density of food outlets available. The highest proportions who said that they regularly purchased something outside of school at lunchtime were from Sch05 (92.4%) and Sch01 (90.0%), two of the most socio-economically deprived of those that took part (with a high and moderate density of food outlets, respectively). 

On the day the PRQ was administered (see Table 4), 53.6% of students said they purchased at least one food or drink item outside school at lunchtime. Across schools, this ranged from around one quarter of students at Sch06 (high SES, low food outlet density) to two thirds in some of the schools (Sch01 low SES, moderate food outlet density; Sch05 low SES, high food outlet density; Sch04 mixed SES, moderate food outlet density) 

Young people shopped at a variety of outlets at lunchtime. The most popular outlets where purchases were made were takeaways, chip shops or fast food outlets (25.8%); newsagents and sweet shops (25.1%); supermarkets (23.0%); and grocery or corner shops (20.1%). Sixteen percent of young people purchased something from a sandwich shop or bakery and 11.1% bought from a burger, chip or ice cream van. No discernible patterns according to SES were found though the availability of outlets was, not surprisingly, related to differences in purchasing from each outlet category at each school.

The most commonly reported food items purchased outside school at lunchtime were chips (26.1%); hot or cold sandwiches, filled rolls or baguettes (23.9%); sweets (21.4%); chocolate (20.2%); and crisps or similar snacks (19.3%). Few students said they purchased fruit (4.2%) or salad (1.7%) outside school at lunchtime. The questionnaire data show that most young people purchasing chips did so from independent rather than chain outlets. The PRQ data also shows that many young people who purchased chips consumed them with other high fat or high salt items such as cheese, curry sauce, fried rice or gravy. Schools where there were more outlets selling items such as chips nearby were those where a higher proportion of pupils reported purchasing chips at lunchtime—Sch01 (37.8%) (low SES, moderate food outlet density) and Sch05 (40.5%) (low SES, high food outlet density). A lower proportion of all reported food purchases were chips at Sch02 (6.9%) (low SES, low food outlet density) and Sch07 (3.6%) (mixed SES, high food outlet density), where chip shops and takeaways were less likely to be available nearby. Sweets represented a high proportion of all food purchases reported by pupils from Sch01 (35.6%) (low SES, moderate food outlet density), Sch02 (44.8%) (low SES, low food outlet density) and Sch03 (25.8%) (low SES, moderate food outlet density).

Fieldwork observations and focus group data suggest that very few students bought food to share at lunchtime; few bought large “sharing” bags of sweets, for example. The schools where a higher proportion of young people reported purchasing sweets overall were often those where food outlets sold unpackaged sweets, either through “pick and mix” selections in supermarkets or convenience stores or from ice cream vans that sold individually priced sweets from larger bags. This meant young people could spend very little but still purchase sweets.

The most commonly purchased drinks among young people who bought one outside school at lunchtime (*n* = 200) were regular sugar sweetened drinks (*n* = 84; 42.0%) and energy drinks (*n* = 67; 33.5%). The number of young people reporting purchasing energy drinks and regular sugar sweetened drinks beyond the school gate at lunchtime represents 28.2% (*n* = 151) of all pupils who completed the PRQ (not just those who reported purchasing a drink). 

Drink purchases were bought from a variety of outlet types though it was notable that at one of the low SES schools (Sch02) drinks were likely to be bought from ice cream vans (this school had a low food outlet density, so choice was limited). Many pupils from Sch02 and Sch03 (both low SES schools, with a low and moderate food outlet density respectively) reported not consuming much food before school or during mid-morning break. The PRQ data show, for example, that a commonly observed pattern in the data for a pupil reporting a sugar sweetened drink or energy drink purchase at lunchtime at Sch02 was a cup of tea before school; water at mid-morning break and an energy drink for lunch (with no food consumption or purchase reported).

### 3.5. Reasons for Buying Food and Drink Beyond the School Gate at Lunchtime

In the PRQ, young people were asked to rate the importance of 19 factors in relation to all the food and drink they purchased outside school on the day the questionnaire was administered (see Table 5). Taste was rated as the most important factor by far, with 97.5% of respondents agreeing that taste was important when they selected what to purchase beyond the school gate; the ingredients or component elements of a product (such as a sandwich) were also considered important by almost three quarters of participants (72.8%). Table 5 highlights that young people at the school with high SES (Sch06) were less likely than other young people to rate taste or ingredients as important. Price, offers and discounts were rated as important by pupils overall (88.9%, 60.9%, and 60.5%, respectively). Young people at Sch06 (high SES; low food outlet density) were particularly unlikely to rate advertisements as a factor affecting their purchasing habits. 

Taste and price were factors that also frequently emerged from the qualitative data analysis. For example, there were many comments from students about purchasing food or drink outside school such as “*you can have more for the price [you] paid or even more variety”* (Sch02) and *“I only had £1 and the food is really nice*” (Sch05). 

The qualitative analysis highlighted the main reasons for students to leave school at lunchtime. The reasons given indicated a negative perception about the food and drinks offered in schools, including not feeling satisfied with the food offered, poor quality food and service, high prices, poor value for money, disliking the (prepaid card) system of payment, queuing for food and the overall environment or the eating environment in school. For example, pupils reported:

“The school never put the tables out anymore (in the canteen) so there is nowhere to sit”

(Sch02, low SES, low food outlet density)

“You have to have exact money in school, which is annoying”

(Sch03, low SES, moderate food outlet density)

“If I was to stay in school all the time…I’d spend like a lot more money than I do outside school…the pizza is like £4, the popcorn is 60p, the juice is 25p, that’s like £4.50… so I’m better going to the shop.”

(Sch07, mixed SES, high food outlet density)

The food and drink offered in schools was rarely seen as healthy as well as “tasty”, with fruit seen as very poor quality for example (“*disgusting”, “all squishy*” (Sch04; mixed SES, moderate food outlet density)). Some students commented that healthy options should be available within schools, yet this was not always perceived to be the case (“*the school want [us] to eat healthy and then you go there and there is pizza and cookies’* (Sch06; high SES, low food outlet density)). Young people at Sch06, the least deprived of our schools, were most likely to comment on the (un)healthiness of the food and drink available to them and this was the only school where young people said they talked with parents about whether their lunchtime practices were healthy or not. The Kitchen Supervisor and Head Teacher at this school said that parents phoned them to discuss their children’s diets or to ask for advice about encouraging more nutritious choices, the only school where this was reported. 

Table 6 highlights that from the eight factors included in the PRQ about reasons to visit specific places to purchase food or drink, young people were more likely to report going somewhere because their friends go there (88.9 %) or the outlet was close to their school (87.3%). Less important but still a consideration for more than half of participants were the atmosphere of the outlet (58.2%) and the availability of special offers and meal deals (57.8%). 

The qualitative data provide further, more nuanced insights. Students who reported going outside school at lunchtime said they did so for a range of reasons, including to consume specific food and drink items not readily available in school (such as chips, salt and sugar-sweetened drinks), and to access food and drink at lower prices; lower prices were perceived as offering value for money. Prices were often discounted for school children at independent outlets, facilitating their purchasing habits. Most students wanted to buy what they considered as good quality food, for example there were comments about food that was too greasy being available beyond the school gate, meaning these young people shopped around to find food that suited their tastes.

*“[Food] at the Spar shop…the hot food is too greasy. It makes my hands feel horrible. Like when you bite into it and you get the oil”*

(Sch07; mixed SES, high food outlet density)

Young people also said that they like to take a break during the school day and like to get fresh air at lunchtime. A desire to spend time with friends was more likely to be achieved by leaving school, since there were more places to socialise and young people did not feel rushed; in school, where catering staff wanted the cafeteria to be cleared as soon as food was consumed, participants felt less comfortable.

*“The girl who owns the cafeteria or whatever, she always chucks you out”*

(Sch04; mixed SES, moderate food outlet density)

Almost three-quarters (73.8%) of students completing the PRQ agreed that the service they received in outlets beyond the school gate was important and the qualitative data also suggest that service was an influential factor. Around schools of low SES many independent retailers knew the students by name, and they worked hard to develop a good customer/retailer relationship.

*“It’s a family run business…Everybody knows everybody…even the kids when they start school and their mother as well, come in here, even in the evening”*

(Retailer close to Sch04; mixed SES, moderate food outlet density)

Conversely, poor service and an unwelcoming attitude emerged from the qualitative data as factors deterring some (though not all) young people from electing to purchase from some food businesses.

*“There is too much queue (at the burger van), they push you and sometimes the women working there get angry”*

(Sch02; low SES, low food outlet density)

Whilst the findings in Table 6 suggest that the proximity of food outlets to a school was important (87.3%), this was not borne out strongly by the qualitative analysis. Very few students mentioned distance as being important. Young people were as likely to say that they were prepared to travel *further* to get the food or drink they wanted, rather than going to places that were particularly close. Students from Sch01, Sch02, Sch03 and Sch04 were observed by the research team running further than 800 m to buy specific food items or to save money by shopping “further afield” (these schools had a low or moderate food outlet density nearby). Shortening the lunch break to prevent young people frequenting external food outlets was not a successful strategy; young people still managed to buy food and drink at external outlets, including before and after school. 

## 4. Discussion

The findings show that more than 90% of young people at some of the deprived schools in the study were leaving school regularly at lunchtime, compared with only four in ten pupils from the least deprived school. There is therefore an ongoing obligation within public health to better understand the nuanced ways that SES informs young people’s lived experience of food and drink purchasing [29]. Family SES cannot be un-entangled from the habits of young people and other studies show the embedded ways that social capital, “fitting in” and deep-rooted perceptions of “functionality over form” (i.e. the need to eat versus the need to eat “well”) structure food and eating [30,31] and this literature needs to inform future intervention with young people at school. Perceptions among young people from varying socio-economic backgrounds about what makes a socially acceptable diet, school dining environment or good relationships with school peers, adults and retailers must be more fully investigated and understood to address pervasive poor nutrition and dietary outcomes. 

The findings show that many young people prioritise wanting “tasty” food at prices that they feel offer value for money at lunchtime. They want to be with their friends, and they take note of the service they receive; some are prepared to shop further than 800 m from school in order to buy food or drink that meets these needs. Whilst the questionnaire data suggest proximity of a food outlet to schools is something young people feel is important when making a purchase, the qualitative findings add to the evidence from other studies that show purchasing is not clearly or solely linked to the density or proximity of food retailers [32]. A narrow focus on the external food environment, in terms of future research, intervention or regulation may not alone reap significant public health gains. 

Whilst schools have a tight budget to work within and, in some countries, have to meet nutrition standards, it is important that schools engage young people to help develop an appropriate eating environment and sell food items that young people want to consume [10,33]. This means, for example, paying attention to portion size, so that food is seen to offer value for money in comparison to what is readily available from commercial food outlets. This does not mean ignoring nutrition standards but ensuring that the food served satiates young people’s appetites at a price they find acceptable. Service must also meet the standards that young people feel they receive on the high street, where retailers work hard to develop relationships with their young customers [26,27,34]. Unfriendly food environments (outside mixed SES schools) alongside feeling unwelcome within the school cafeteria (low SES schools) were socio-cultural aspects of lunchtime, driven by the socio-economic status of an area, which influenced whether young people left school at lunchtime to buy and consume food or drink. Indeed, the highest proportion of pupils who said that they regularly purchased something outside of school at lunchtime and, on the day the PRQ was administered, they purchased at least one food or drink item outside school were the students from low and mixed SES schools. There is a clear need for effective ways to engage such young people, their teachers and their caterers in holistically addressing the dining environment to overcome these barriers to eating in school. 

The way that young people perceive and value the “healthiness” of the food sold to them in schools must not be taken for granted; whilst in this study young people from the least deprived school were most likely to discuss health and their parents seemed more engaged in wanting to ensure their child ate a healthy diet, that is not to say that other young people disregard the nutritional quality of their diet [5,35]. Responses to “healthy eating” discourses are socially constructed; young people understand the moral value attached to whether they undertake “authentic”, i.e., government sanctioned healthy eating practices, and this, together with the SES of their family, influences the healthiness of their dietary decisions [36,37]. There is, therefore, more to be done to inform and meaningfully engage young people in debates about healthy eating. Appealing to their desire to eat tasty and at the same time healthy food is one way to achieve this. Whilst parents and teachers could be involved in such interventions their input and whether they are considered positive role models by young people cannot be guaranteed [11]; innovative strategies to effectively work with teenagers are therefore called for. Other studies show the multiple challenges involved in achieving this but also point the way for approaches to be co-designed by and with young people and the avoidance of “top down” assumptions about what pupils “should” engage with [38,39]. For example, many young people have a limited experience of trying some (even commonly available) “healthier” foods such as fruits or vegetables therefore adults have a responsibility to find ways to tempt them to try and experiment with new flavours to encourage a healthier way of eating. One initiative that is taking this approach is Flavour School, which provides sensory food education in the UK based on a successful model used elsewhere in Europe [40]. Universal initiatives are less likely to be successful than ones that are targeted at particular groups [11,32] as our findings suggest that young people attending a high SES school might prioritise different factors than their peers from other socio-economic groups, who are more likely to rate taste, ingredients and advertisements as important. Thus, interventions must consider the SES of the school, the characteristics of the pupils and the nature of the local food environment (considering the availability and accessibility of food and drink options). 

There is also a need to consider what young people eat at lunchtime within the broader context of the whole school day. Our findings show that two thirds of young people eat something before school (though 17% did not, rising to a quarter at some schools) and two-thirds had something to eat at mid-morning break. A quarter of participants did not eat lunch on the day they were questioned, but if they ate at earlier time points this is perhaps less of a worry in terms of overall energy intake; concern should perhaps focus on the young people who eat at *no* point during the school day as well as those who are regularly (rather than occasionally) consuming food or drinks high in fat, sugar or salt during the school lunch break by purchasing beyond the school gate. It is unrealistic to expect young people to completely avoid food high in fat, sugar or salt when shopping within the commercial sector, since energy dense food and drink is so readily available and at a cost that appeals to teenagers (and not just from fast food/takeaway outlets) [41]. Challenging young people to reduce the number of times they go outside school at lunchtime is possibly a more effective strategy, particularly if combined with initiatives to improve the food and service offered within schools, to encourage students to stay on site [34]. 

One strength of this study is its use of a mixed-methods design; this allowed insights about young people’s food and eating practices to come to the fore. SES was investigated from a different viewpoint than is usual in studies of socio-economic deprivation, drawing on the local environment in relation to food outlets and trying to explore the lived experience of SES during the school day. With regard to potential limitations of this study, we were unable to include a greater number of high SES schools because of time constraints. Using focus groups was limiting in terms of asking young people about their experiences of low family income, in relation to being eligible for free school meals for example, as there is stigma and shame attached to this welfare benefit meaning some young people were not able to voice their opinion openly. We will use a different method in future studies to overcome this, particularly as the issue of hunger and poverty in childhood is a public health concern in the UK.

## 5. Conclusions

Practices relating to food and eating are complex phenomena that are intricately woven and informed by socio-economic factors. This study benefited from using a mixed-methods design to reveal important insights that can inform the development of interventions to enhance young people’s food practices, achieve public health gains and address socio-economic inequalities. This approach is therefore recommended in further research [32] that seeks to inform intervention to improve young people’s health, wellbeing and nutritional status [42]. It is imperative that evidence from studies such as this is utilised by multi-sectoral teams involving education departments, school staff, public health colleagues, academics, caterers, and importantly, young people. To ignore evidence from studies that clearly show the important and embedded ways that SES informs the food practices of young people is, in our view, immoral. Families and young people cannot be held to account for what they eat without greater reference to the way that socio-economic inequalities develop and persist. 

## Figures and Tables

**Table 1:** Number of young people who participated in the study.

School	Individual and Group Interviews	Go-Along Tours	Focus Groups	Semi-Structured Written Activity	Total Participants in Qualitative Study	Purchasing Recall Questionnaire
**Sch01**	7	7	21	41	**76**	80
**Sch02**	8	0	20	79	**107**	96
**Sch03**	7	3	22	57	**89**	68
**Sch04**	16	2	24	71	**113**	90
**Sch05**	7	0	35	58	**100**	66
**Sch06**	5	4	20	65	**94**	61
**Sch07**	0	0	13	59	**72**	74
**Total**	**50**	**16**	**155**	**430**	**651**	**535**

**Table 2:** Participating schools’ information.

School	No of Students on School Roll	SIMD Category ^a^	% FSM ^b^	SES	Number of Food Outlets within 800 m of School ^c^
**Sch01**	<600	1 (most deprived)	30–40	Low	21–99
**Sch02**	>1000	1 (most deprived)	10–20	Low	0–20
**Sch03**	<1000	1 (most deprived)	20–30	Low	21–99
**Sch04**	<1000	3 (least deprived)	20–30	Mixed	21–99
**Sch05**	<600	1 (most deprived)	20–30	Low	100+
**Sch06**	>1000	3 (least deprived)	0–10	High	0–20
**Sch07**	<600	2 (moderately deprived)	30–40	Mixed	100+

^a^ 1 = SIMD ranks 1–2602 (four most deprived deciles); 2 = SIMD ranks 2603–3903 (two middle deciles); 3 = SIMD ranks 3904–6505 (four least deprived deciles). % registered for free school meals (FSM) at the school based on the 2013 FSM dataset. ^b^ The proportion of pupils registered for FSM at secondary schools across Scotland is 15.5% (this includes pupils attending local authority and grant-maintained schools). ^c^ The number of food outlets was categorised as indicating a high (100+), moderate (21–99) or low (0–20) outlet density, for ease of referring to the food environment in the narrative.

**Table 3:** Total number of registered and observed food businesses, by outlet type, within 800 m of schools.

	Number and Category of Food Outlet (within 800 m)									
	Take-Away	Sports/Leisure Centre	Petrol Station	Food Vehicle	Bakery	Super-Market/Grocer	News-Agent/Post Office	Café/Restaurant	Other	Total
**Sch01**	12	0	1	0	2	6	2	6	22	**51**
**Sch02**	0	1	0	2	1	2	2	4	4	**16**
**Sch03**	8	0	0	4	1	1	5	4	8	**31**
**Sch04**	10	0	0	1	2	5	2	0	13	**33**
**Sch05**	19	2	0	1	6	11	11	50	149	**249**
**Sch06**	1	0	0	0	1	0	2	0	1	**5**
**Sch07**	13	3	0	2	2	10	7	23	44	**104**
**Total**	63	6	1	10	15	35	31	87	241	**489**

**Table 4:** Students purchasing food/drink outside school at lunchtime on the day they completed the Purchasing Recall Questionnaire.

Food or Drink Purchased	All	Sch01	Sch02	Sch03	Sch04	Sch05	Sch06	Sch07
**Yes**	287 (53.6)	54 (67.5)	43 (44.8)	36 (52.9)	61 (67.8)	43 (65.2)	14 (23.0)	36 (48.6)
**No**	248 (46.4)	26 (32.5)	53 (55.2)	32 (47.1)	29 (32.2)	23 (34.8)	47 (77.0)	38 (51.4)
**TOTAL**	535 (100.0)	80 (100.0)	96 (100.0)	68 (100.0)	90 (100.00)	66 (100.0)	61 (100.0)	74 (100.0)

**Table 5:** Factors important when food/drink purchased at lunchtime.

	All (*N* = 243)	Sch01 (*N* = 43)	Sch02 (*N* = 36)	Sch03 (*N* = 28)	Sch04 (*N* = 54)	Sch05 (*N* = 42)	Sch06 (*N* = 11)	Sch07 (*N* = 29)
**Taste**	237 (97.5)	42 (97.7)	34 (94.4)	28 (100.0)	54 (100.0)	42 (100.0)	9 (81.8)	28 (96.6)
**Price**	216 (88.9)	38 (88.4)	33 (91.7)	28 (100.0)	49 (90.7)	36 (85.7)	10 (90.9)	22 (75.9)
**Ingredients**	177 (72.8)	29 (67.4)	27 (75.0)	23 (82.1)	42 (77.8)	29 (69.0)	6 (54.5)	21 (72.4)
**Price discount**	148 (60.9)	29 (67.4)	21 (58.3)	13 (46.4)	40 (74.1)	23 (54.8)	5 (45.5)	17 (58.6)
**Product on offer (meal deal/BOGOF)**	147 (60.5)	28 (65.1)	25 (69.4)	14 (50.0)	36 (66.7)	24 (57.1)	5 (45.5)	15 (51.7)
**Brand**	139 (57.2)	24 (55.8)	22 (61.1)	14 (50.0)	29 (53.7)	29 (69.0)	4 (36.4)	17 (58.6)
**Packaging**	132 (54.3)	21 (48.8)	23 (63.9)	14 (50.0)	34 (63.0)	23 (54.8)	5 (45.5)	12 (41.4)
**It is easy to grab**	117 (48.1)	21 (48.8)	18 (50.0)	16 (57.1)	28 (51.9)	15 (35.7)	4 (36.4)	15 (51.7)
**One of the 1st products I see**	112 (46.1)	22 (51.2)	14 (38.9)	16 (57.1)	22 (40.7)	18 (42.9)	4 (36.4)	16 (55.2)
**Displays**	106 (43.6)	20 (46.5)	17 (47.2)	14 (50.0)	24 (44.4)	18 (42.9)	3 (27.3)	10 (34.5)
**Brand sponsorship**	93 (38.3)	14 (32.6)	14 (38.9)	14 (50.0)	20 (37.0)	14 (33.3)	4 (36.4)	13 (44.8)
**It is close to the till**	92 (37.9)	16 (37.2)	15 (41.7)	15 (53.6)	18 (33.3)	12 (28.6)	3 (27.3)	13 (44.8)
**Television Adverts**	91 (37.4)	18 (41.9)	14 (38.9)	11 (39.3)	19 (35.2)	18 (42.9)	0 (0.0)	11 (37.9)
**Celebrities endorsement**	81 (33.3)	13 (30.2)	12 (33.3)	13 (46.4)	18 (33.3)	14 (33.3)	2 (18.2)	9 (31.0)
**Online Adverts**	79 (32.5)	12 (27.9)	13 (36.1)	12 (42.9)	19 (35.2)	12 (28.6)	0 (0.0)	11 (37.9)
**Other Adverts**	79 (32.5)	14 (32.6)	14 (38.9)	10 (35.7)	20 (37.0)	10 (23.8)	1 (9.1)	10 (34.5)
**Cartoon endorsement**	79 (32.5)	12 (27.9)	13 (36.1)	13 (46.4)	19 (35.2)	9 (21.4)	2 (18.2)	11 (37.9)
**Chance to win free things**	77 (31.7)	14 (32.6)	16 (44.4)	9 (32.1)	17 (31.5)	11 (26.2)	1 (9.1)	9 (31.0)
**Online interactive games**	74 (30.5)	12 (27.9)	10 (27.8)	9 (32.1)	19 (35.2)	12 (28.6)	2 (18.2)	10 (34.5)

**Table 6:** Reasons for visiting outlets beyond the school gate on day the Purchasing Recall Questionnaire was administered.

	All (*N* = 244)	Sch01 (*N* = 43)	Sch02 (*N* = 36)	Sch03 (*N* = 28)	Sch04 (*N* = 54)	Sch05 (*N* = 42)	Sch06 (*N* = 12)	Sch07 (*N* = 29)
**My friends go to this place**	217 (88.9)	38 (88.4)	30 (83.3)	25 (89.3)	52 (96.3)	35 (83.3)	11 (91.7)	26 (89.7)
**It is close to my school**	213 (87.3)	35 (81.4)	30 (83.3)	26 (92.9)	51 (94.4)	34 (81.0)	11 (91.7)	26 (89.7)
**I like the quality**	207 (84.8)	38 (88.4)	29 (80.6)	25 (89.3)	46 (85.2)	36 (85.7)	11 (91.7)	22 (75.9)
**I like the variety**	204 (83.6)	36 (83.7)	30 (83.3)	24 (85.7)	48 (88.9)	35 (83.3)	9 (75.0)	22 (75.9)
**I like the service**	180 (73.8)	35 (81.4)	26 (72.2)	22 (78.6)	44 (81.5)	29 (69.0)	7 (58.3)	17 (58.6)
**I like the prices**	173 (70.9)	30 (69.8)	24 (66.7)	23 (82.1)	41 (75.9)	31 (73.8)	7 (58.3)	17 (58.6)
**I like the atmosphere**	142 (58.2)	24 (55.8)	26 (72.2)	16 (57.1)	30 (55.6)	27 (64.3)	5 (41.7)	14 (48.3)
**I like the meal deals and special offers**	141 (57.8)	19 (44.2)	17 (47.2)	17 (60.7)	36 (66.7)	30 (71.4)	6 (50.0)	16 (55.2)

## Data Availability

The questionnaire developed for this study is available from the first author. The non-attributable data collected is being considered for deposit in the University of Hertfordshire data repository. Please contact the first author for further details and to request access to the data. Data that reveals the identity of the participating schools and their location will not be made publicly available.
